# Sexual Mania in Elephants

**Published:** 1883-01

**Authors:** 


Sexual Mania in Elephants.—Elephants which are confined in menageries and gardens are subject at certain periods to attacks of sexual excitement which may end in actual mania. Young elephants as they reach maturity and begin to feel the sexual passion show these symptoms oftenest. In their free condition doubtless there is nothing abnormal in it, but in captivity, owing to lack of opportunity to gratify the sexual appetite, the normal excitement produces a chronic nervous disturbance, which is interrupted by period of acute maniacal excitement.

When this condition is coming on the elephant is restless and uneasy. The cheek-glands pour out a large amount of secretion, the eyes have a peculiar look; the animal often trumpets and flaps his ears noisily; the penis is frequently erected and in some cases (Dekker) the animal uses his trunk to masturbate.

In female elephants this condition is rarely so strongly developed.

In all cases the animal must be carefully watched, as he is treacherous and apt to be violent.

The conditions of sexual excitement and mania may be cured in most cases by low diet. Medicine seems to have very little effect upon elephants. Twenty pounds of Glauber's salts and a quarter of a pound of calomel have been given at one time without effect. Dr. Max Schmidt of Frankfort Germany, has given camphor in doses of a quarter of a pound with doubtful benefit. Fluid butter in three pound doses is recommended by Von Hügel.

